# Supplementary material for: Identification of metabolic vulnerabilities of receptor tyrosine kinases-driven cancer
Source: Nat Commun. 2019 Jun 20;10:2701. doi: 10.1038/s41467-019-10427-2 (PMC6586626; doi:10.1038/s41467-019-10427-2)
Supplement: Supplementary file 1 — Supplementary Information [file 41467_2019_10427_MOESM1_ESM.pdf]

Supplementary Information for

Identification of metabolic vulnerabilities of receptor tyrosine kinases-driven cancer

Jin et al.

7 **Supplementary Figure 1**

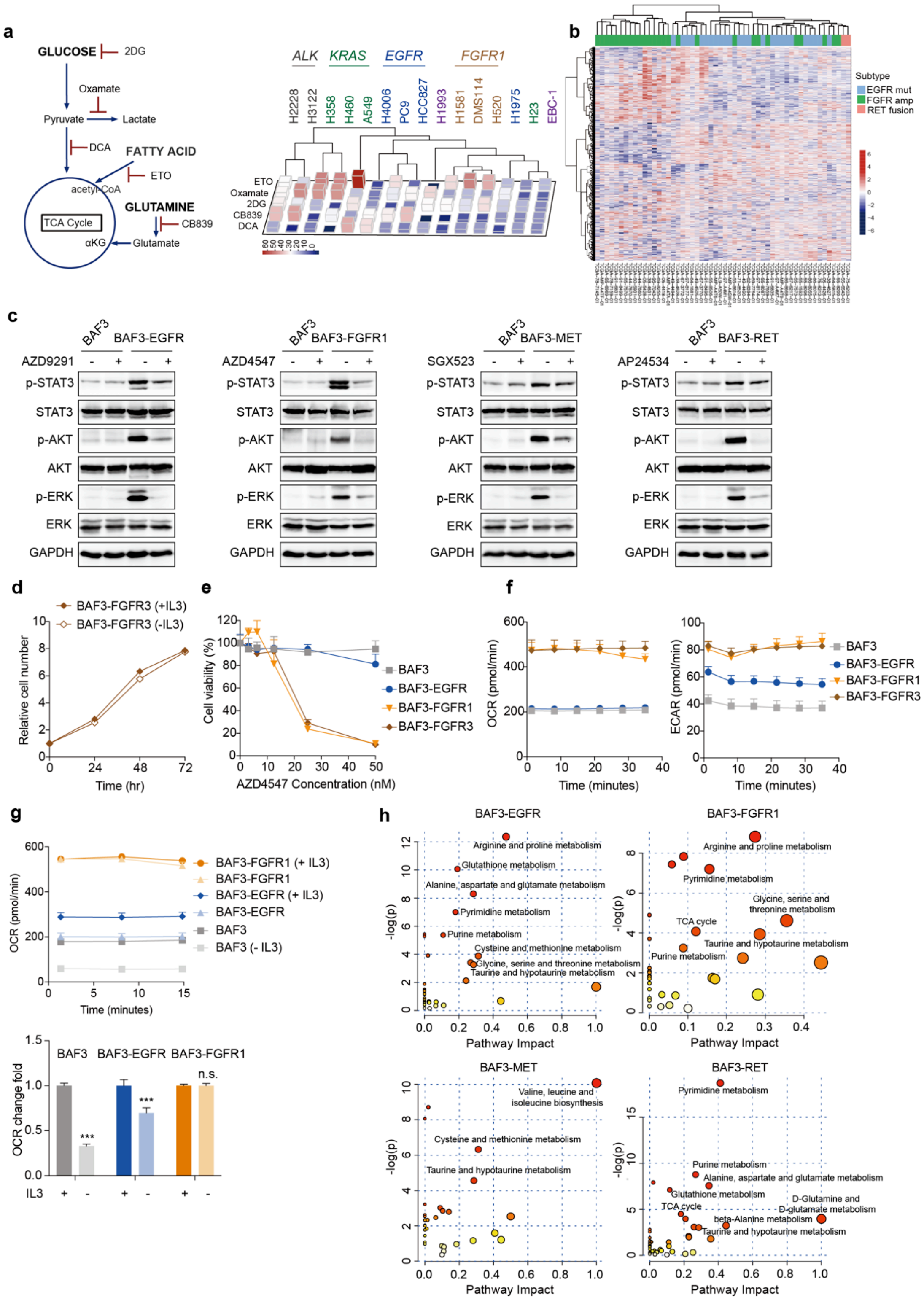

8

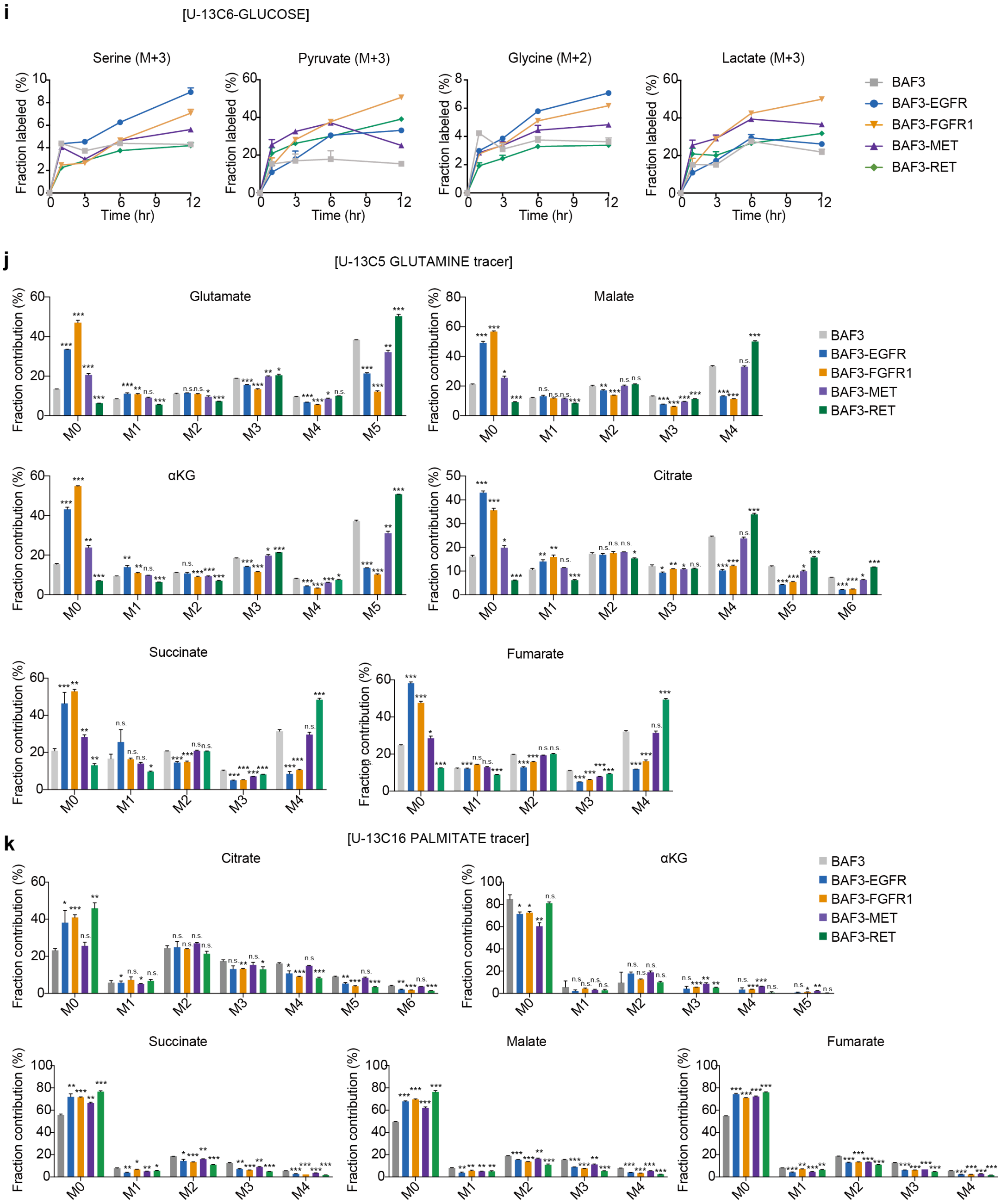

i
[U-13C6-GLUCOSE]
Serine (M+3)
Pyruvate (M+3)
Glycine (M+2)
Lactate (M+3)
Fraction labeled (%)
Time (hr)
BAF3
BAF3-EGFR
BAF3-FGFR1
BAF3-MET
BAF3-RET
j
[U-13C5 GLUTAMINE tracer]
Glutamate
Malate
αKG
Citrate
Succinate
Fumarate
Fraction contribution (%)
M0
M1
M2
M3
M4
M5
M6
k
[U-13C16 PALMITATE tracer]
Citrate
αKG
Succinate
Malate
Fumarate
Fraction contribution (%)

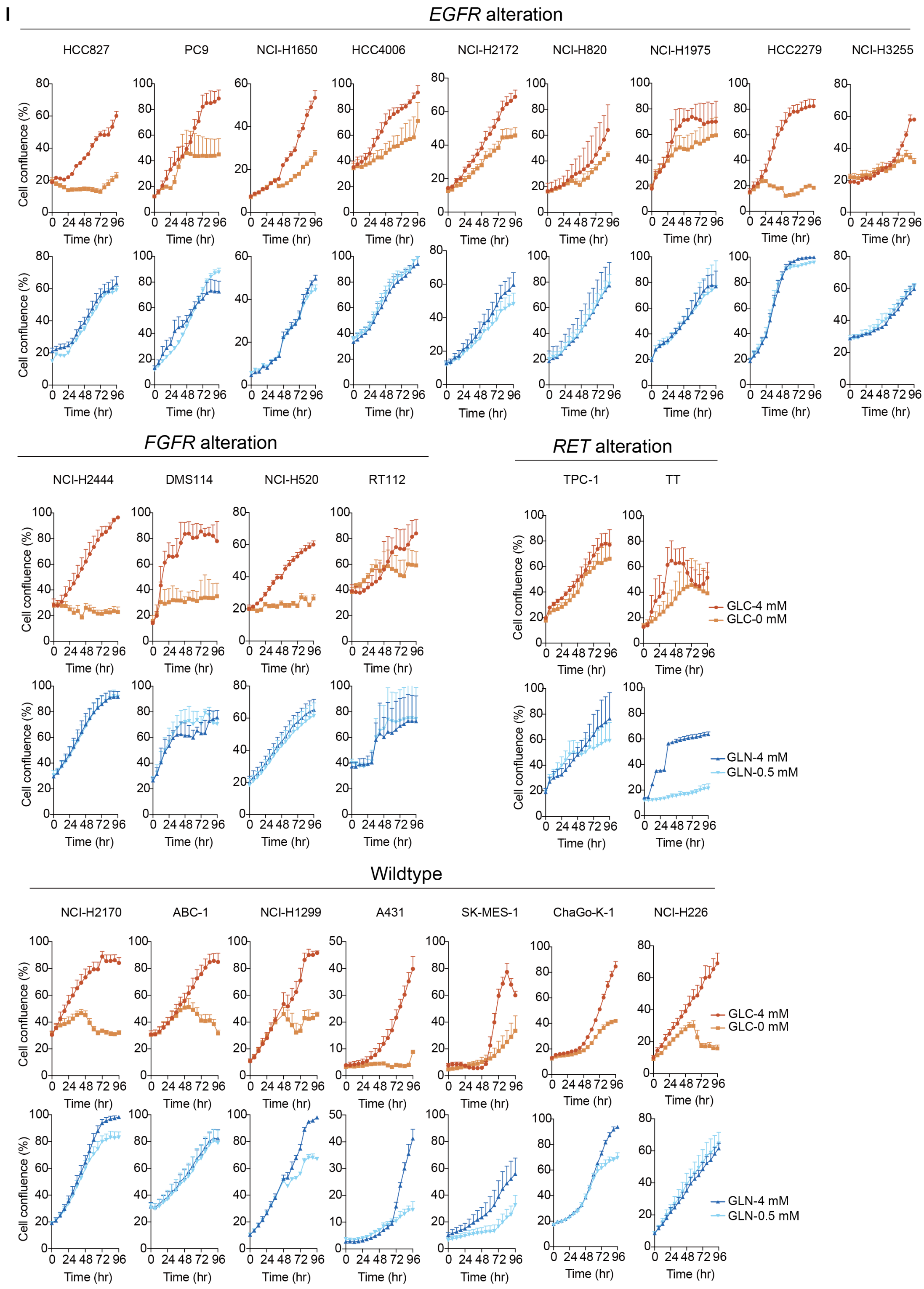
I
EGFR alteration
HCC827
PC9
NCI-H1650
HCC4006
NCI-H2172
NCI-H820
NCI-H1975
HCC2279
NCI-H3255
FGFR alteration
NCI-H2444
DMS114
NCI-H520
RT112
RET alteration
TPC-1
TT
Wildtype
NCI-H2170
ABC-1
NCI-H1299
A431
SK-MES-1
ChaGo-K-1
NCI-H226
Cell confluence (%)
Time (hr)
GLC-4 mM
GLC-0 mM
GLN-4 mM
GLN-0.5 mM

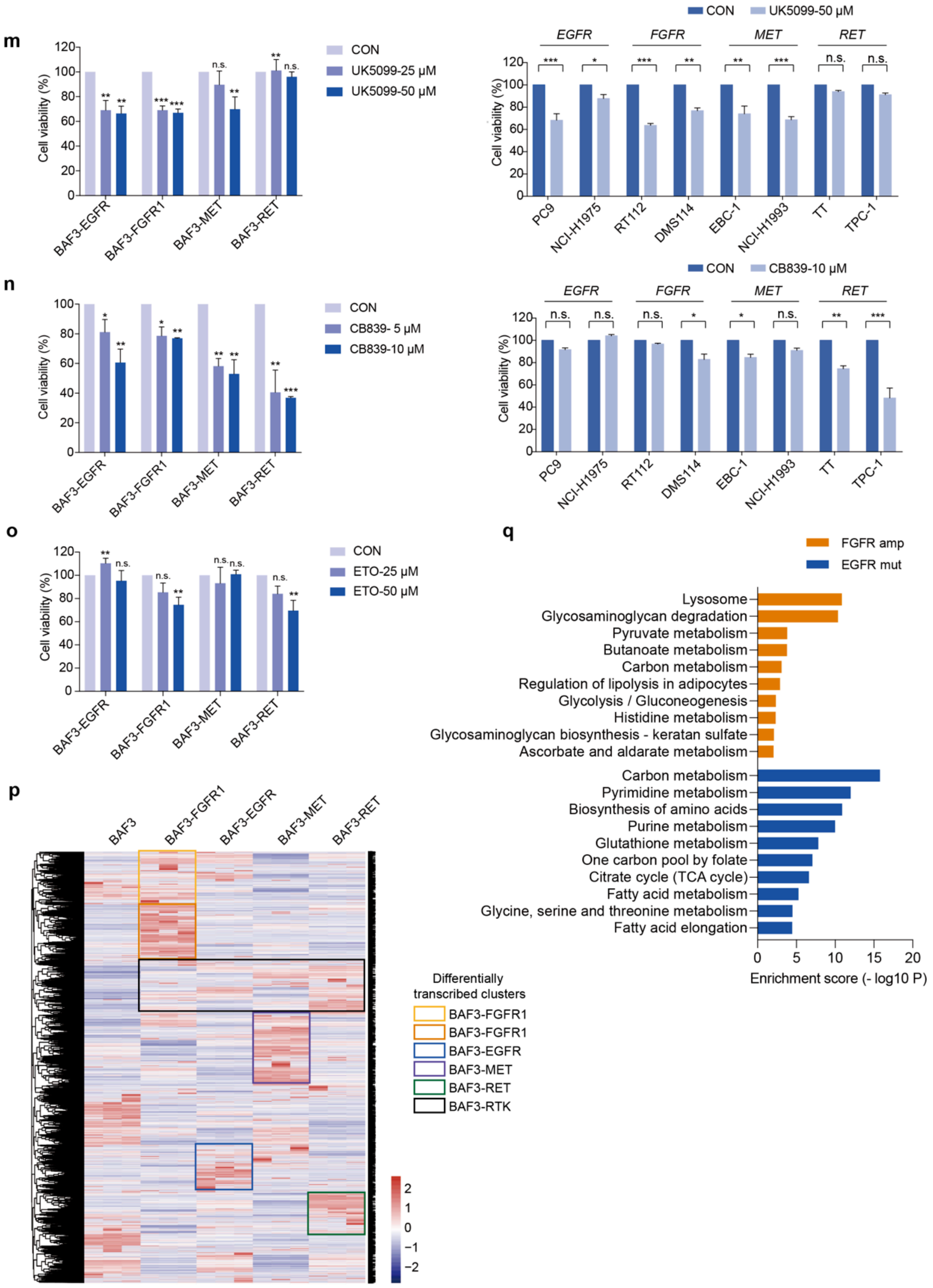
m
Cell viability (%)
CON
UK5099-25 μM
UK5099-50 μM
BAF3-EGFR
BAF3-FGFR1
BAF3-MET
BAF3-RET
CON
UK5099-50 μM
EGFR
FGFR
MET
RET
PC9
NCI-H1975
RT112
DMS114
EBC-1
NCI-H1993
TT
TPC-1
n
CON
CB839- 5 μM
CB839-10 μM
CON
CB839-10 μM
o
CON
ETO-25 μM
ETO-50 μM
p
BAF3
BAF3-FGFR1
BAF3-EGFR
BAF3-MET
BAF3-RET
Differentially transcribed clusters
BAF3-FGFR1
BAF3-FGFR1
BAF3-EGFR
BAF3-MET
BAF3-RET
BAF3-RTK
q
FGFR amp
EGFR mut
Lysosome
Glycosaminoglycan degradation
Pyruvate metabolism
Butanoate metabolism
Carbon metabolism
Regulation of lipolysis in adipocytes
Glycolysis / Gluconeogenesis
Histidine metabolism
Glycosaminoglycan biosynthesis - keratan sulfate
Ascorbate and aldarate metabolism
Carbon metabolism
Pyrimidine metabolism
Biosynthesis of amino acids
Purine metabolism
Glutathione metabolism
One carbon pool by folate
Citrate cycle (TCA cycle)
Fatty acid metabolism
Glycine, serine and threonine metabolism
Fatty acid elongation
Enrichment score (- log10 P)

Supplementary Figure 1

a, Sensitivity of metabolism-targeted inhibitors in lung cancer cell lines bearing indicated genetic alterations. Left, Diagram showing the inhibitors used for metabolic pathways intervention. Right, Bar plot obtained by the unbiased hierarchical cluster analysis of the cell lines according to cell growth inhibition rate of individual metabolic inhibitors. Cells were treated with ETO (50 μM), CB839 (10 μM), Oxamate (30 mM), DCA (10 mM) or 2DG (2.5 mM) for 72 hr. Cell growth inhibition rate was measured using the CCK8 assay **b**, Heatmap obtained by cluster analysis using the mRNA data of metabolic genes of 740 lung adenocarcinoma patients in the TCGA data sets. The columns indicate different patients bearing indicated genetic alterations, and the rows indicate different metabolic genes. **c**, Immunoblotting analysis. Cells were treated with indicated RTK inhibitors (100 nM) for 1 hr before being subjected to immunoblotting analysis using indicated antibodies. **d**, IL3 dependence analysis. Cell growth fold changes with or without IL3 were plotted by counting cell numbers. Data were means of triplicates; error bars represented SD. **e**, Cell sensitivity to RTK inhibition. Cells were treated with AZD4547 at indicated concentrations for 72 hr and cell viability was analyzed using CCK8 assay. Data were means of triplicates; error

bars represented SD. **f**, Oxygen consumption rate (OCR) and extracellular acidification rate (ECAR) measurement using Seahorse XF96 analyzer. **g.** The impact of IL3 on OCR. BAF3-RTK or the parental BAF3 cells were cultured in the presence or absence of IL3. OCR were measured using Seahorse XF96 analyzer. Data in **f, g** were means of five replicates; error bars represented SD. **h**, Metabolite set enrichment analysis. The metabolome view showing the representative metabolic pathways arranged by $p < 0.05$ (from pathway enrichment analysis) on Y-axis, and pathway impact > 0.1 (from pathway topology analysis) on X-axis, carried out with MetaboAnalyst 4.0. The node color was based on its p value and the node radius was determined by its pathway impact value. The altered metabolite sets were arranged according to 1.5-fold cutoff and $P < 0.01$ in relative to parental BAF3 cells. **i**, Time courses of ^{13}C labeled intensities of metabolites from [U-$^{13}C_6$]-glucose flux analysis measured by GC/MS. BAF3 and BAF3-RTK cells were cultured in the presence of [U-$^{13}C_6$]-glucose for 1, 3, 6 or 12 hr. The labeling curves were plotted by measuring fractional ^{13}C enrichment at 1, 3, 6 and 12 hr respectively. **j-k**, ^{13}C labeled intensities of metabolites from [U-$^{13}C_5$]-glutamine or [U-$^{13}C_{16}$]-palmitate. BAF3 and

BAF3-RTK cells were cultured for 24 hr in the presence of either [U-$^{13}C_5$]-glutamine or [U-$^{13}C_{16}$]-palmitate. The intermediate metabolites in TCA cycle labeled with ^{13}C isotopologue were measured by GC/MS. Data in **j-k** were means of triplicates; error bars represented SEM. **l**, Glucose/glutamine dependency analysis. Cells were cultured with indicated concentrations of glucose (GLC)/glutamine (GLN) for 4 days. Images were acquired every 6 hr by automated real-time assessment using IncuCyte ZOOM. Growth curves were plotted as the change in confluence percentage. Data were means of six replicates; error bars represented SD. **m-o**, Cell sensitivity to targeted metabolism inhibition. Cells were treated with indicated inhibitors for 72 hr. The viability of BAF3 and BAF3-RTK cells were analyzed using CCK8 assay, and the viability of cancer cells were analyzed using the SRB assay. The assays were performed in biological triplicates, and error bars represented SD. **p**, Transcriptome analysis. Heatmap of transcriptome profiling representing the mRNA levels of genes performed by RNA-seq. The rows indicate different genes, and the columns indicate different cells (n = 3 per cell line). **q**, KEGG pathway enrichment analysis the metabolic genes between EGFR- and FGFR-activated tumors that displayed in **b**. The

significantly enriched metabolism-related KEGG pathways ($P < 0.05$) were presented. For each KEGG pathway, the bar shows the enrichment score of the pathway according to p value. Unless otherwise stated, BAF3 parental cells were cultured with IL3 and BAF3-RTK cells were cultured without IL3. For all bar graphs, $^{***}p < 0.001$, $^{**}p < 0.01$, $^{*}p < 0.05$, n.s. ≥ 0.05 for Student's pairwise t test. Source data are provided as a Source Data file.

Supplementary Figure 2

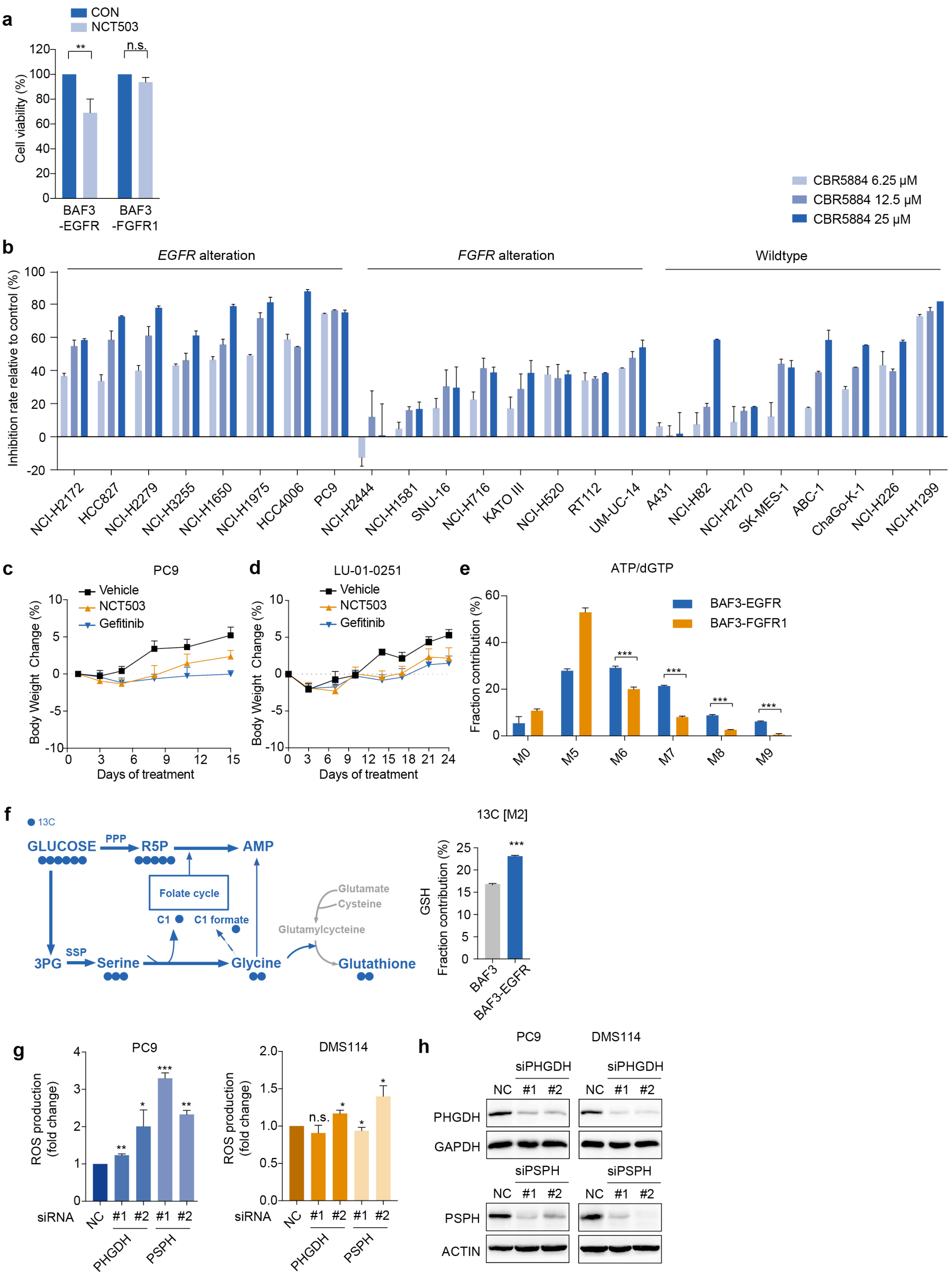

i

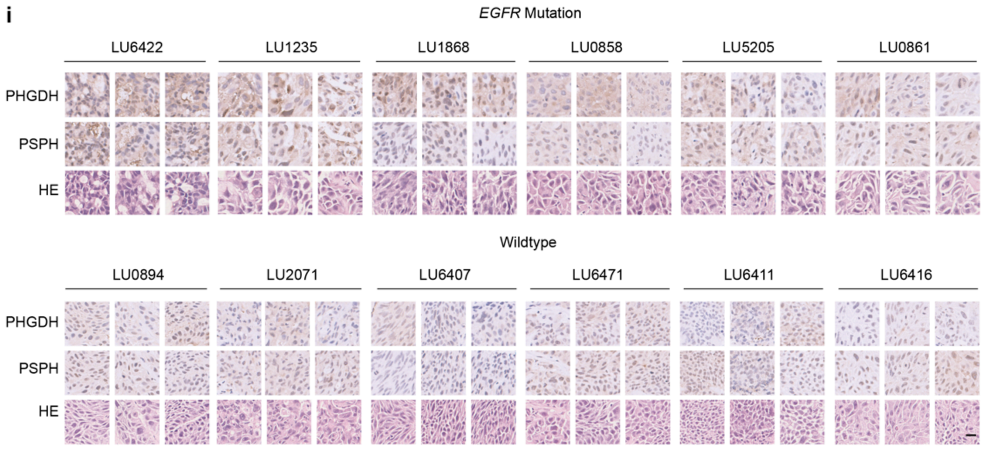

EGFR Mutation
LU6422
LU1235
LU1868
LU0858
LU5205
LU0861
PHGDH
PSPH
HE
Wildtype
LU0894
LU2071
LU6407
LU6471
LU6411
LU6416
PHGDH
PSPH
HE

j

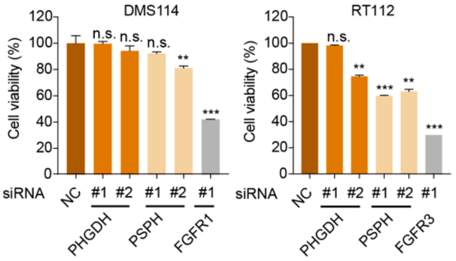

DMS114
Cell viability (%)
120
100
80
60
40
20
0
n.s.
n.s.
n.s.
**
***
siRNA
NC
#1
#2
#1
#2
#1
PHGDH
PSPH
FGFR1
RT112
n.s.
**
***
**
***
FGFR3

k

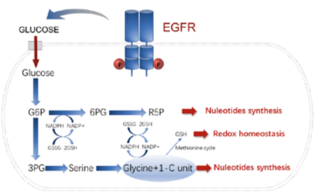

GLUCOSE
EGFR
Glucose
G6P
6PG
R5P
3PG
Serine
Glycine+1-C unit
Nuleotides synthesis
Redox homeostasis
Nuleotides synthesis

Supplementary Figure 2

a, Cell sensitivity to PHGDH inhibition. Cell viability was measured using the CCK8 assay following the treatment with NCT503 (20 μM) for 72 hr. BAF3-EGFR/FGFR1 cells were cultured without IL3. The assays were performed in biological triplicates, and error bars represented SD. **b,** Sensitivity of a panel of cancer cells to PHGDH inhibition. Cancer cells with indicated genetic alterations were treated with CBR5884 at 6.25, 12.5 or 25 μM for 6 days and the inhibition rate of cell growth was determined relative to untreated control. Data were means of duplicates and error bars represented SD. **c, d**, Body weight change of PC9 xenograft and LU-01-0251 PDX models. Mice were dosed with NCT-503 (40 mg/kg) or Gefitinib (5 mg/kg for PC9 and 1 mg/kg for LU-01-0251) daily for indicated days (n = 8 for PC9, n = 6 for LU-01-0251). Data were means and error bars represented SEM. **e**, ^{13}C enrichment of purine nucleotides. BAF3-EGFR/FGFR1 cells were cultured in the presence of [U-$^{13}C_6$]-glucose for 24 hr without IL3. **f**, Tracer scheme illustrating the flux of glucose to glutathione via SSP determined by ^{13}C-labeled metabolites (Left) and the ^{13}C enrichment of glutathione (Right). Cells were cultured in the presence of [U-$^{13}C_6$]-glucose for 24 hr, and the incorporation percentage of ^{13}C from glucose was

analyzed by QTOF-MS. Data were means of triplicates; error bars represented SEM. **g**, ROS level measurement. PC9 and DMS114 cells were transfected with indicated siRNAs for 72 hr. Data were means of duplicates; error bars represented SD. **h**, Immunoblotting analysis. Cells were transfected with indicated siRNAs for 48 hr before being subjected to immunoblotting using indicated antibodies. **i**, Immunohistochemistry analysis of representative tumor tissues from NSCLC PDX tumors with *EGFR* mutation or wildtype RTK. Shown are representative field from one section per tumor tissue (n = 3 independent tumor tissues from each PDX model). Scale bar, 20 μm. **j**, Cell viability assay. DMS114 and RT112 cells were transfected with indicated siRNAs for 72 hr and cell viability was analyzed by counting cell numbers. The assays were performed in duplicates, and error bars represented SD. **k**, Diagram depicting the metabolic reprogramming upon EGFR activation. For all bar graphs, $^{***}p < 0.001$, $^{**}p < 0.01$, $^{*}p < 0.05$, n.s. ≥ 0.05 for Student's pairwise t test. Source data are provided as a Source Data file.

105 Supplementary Figure 3

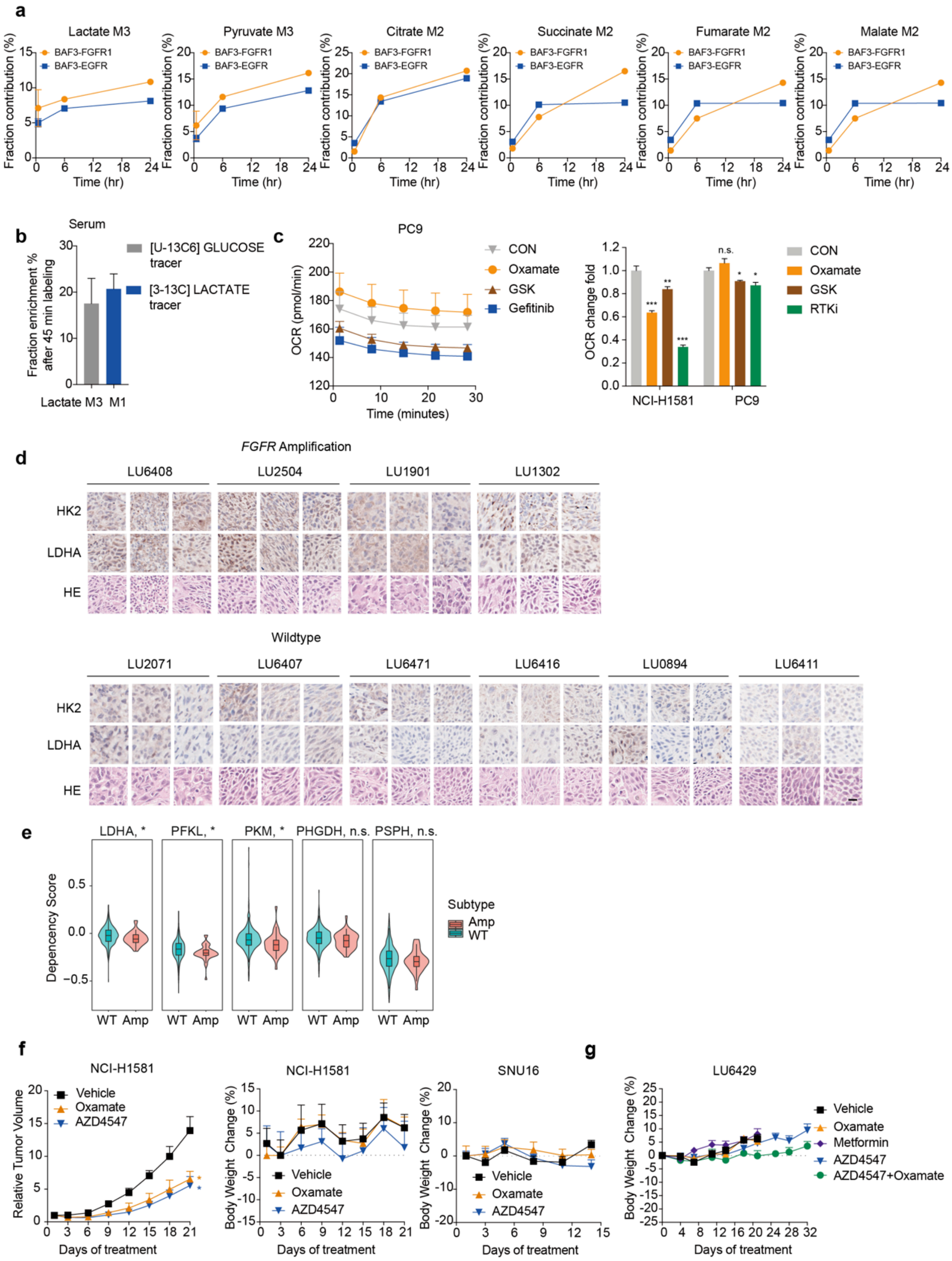

106

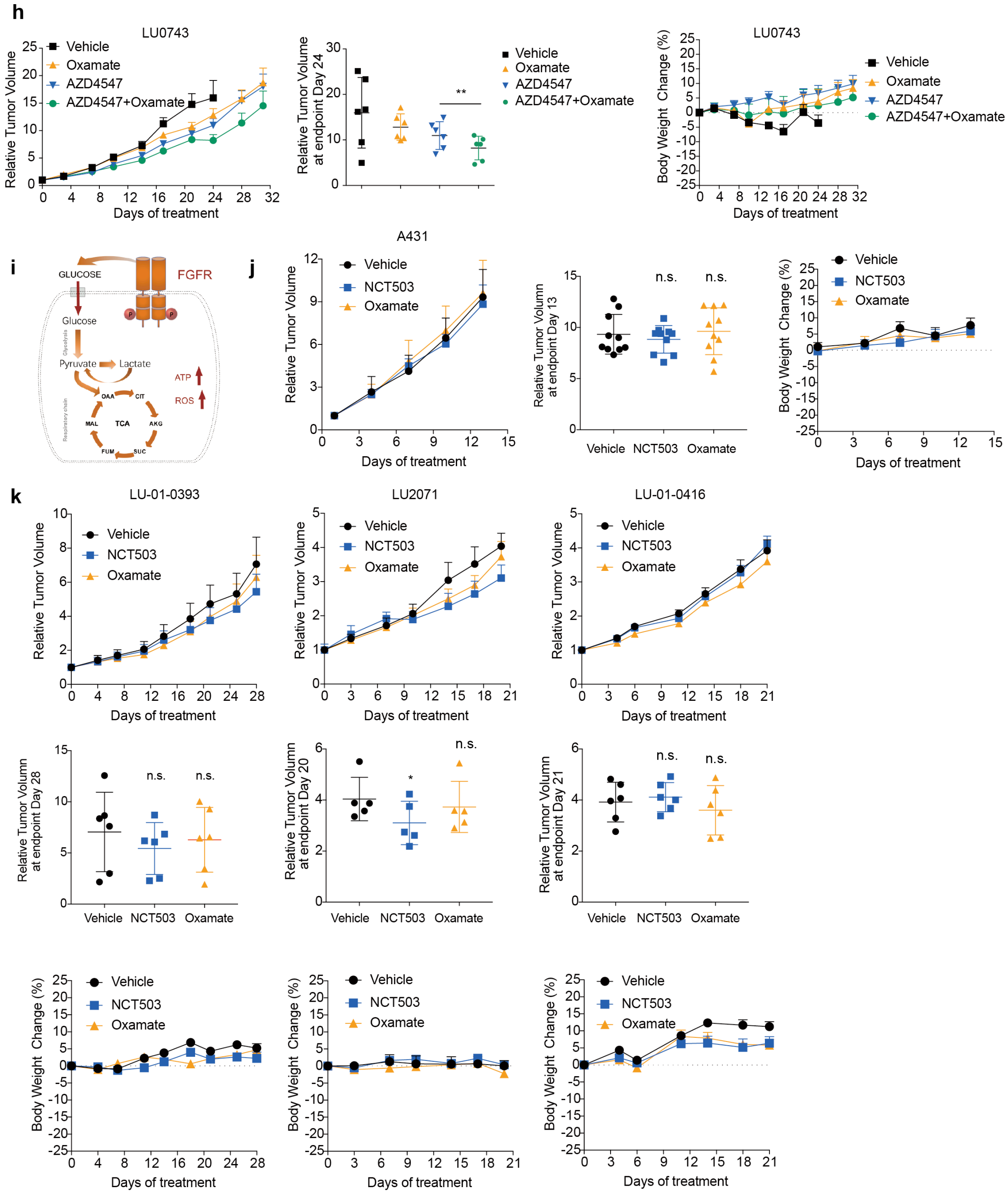
h
LU0743
Vehicle
Oxamate
AZD4547
AZD4547+Oxamate
Relative Tumor Volume
Days of treatment
Relative Tumor Volume at endpoint Day 24
**
Body Weight Change (%)
i
GLUCOSE
FGFR
Glucose
Pyruvate
Lactate
ATP
ROS
OAA
CIT
MAL
TCA
AKG
FUM
SUC
j
A431
Vehicle
NCT503
Oxamate
Relative Tumor Volumn at endpoint Day 13
n.s.
k
LU-01-0393
LU2071
LU-01-0416
Relative Tumor Volumn at endpoint Day 28
Relative Tumor Volumn at endpoint Day 20
Relative Tumor Volumn at endpoint Day 21
*

Supplementary Figure 3

a, Time courses of ^{13}C labeled intensities of metabolites from [U-$^{13}C_3$]-lactate flux analysis measured by GC/MS. The labeling curves were plotted by measuring fractional ^{13}C enrichment at 0.5, 6 and 24 hr respectively. BAF3-EGFR/FGFR1 cells were cultured without IL3. The assays were performed in biological triplicates, and error bars represented SD. **b**, Serum fractional enrichment of lactate. Mice with flank xenografts of H1581 cells were co-injected with [U-$^{13}C_6$]-glucose and [3-^{13}C]-lactate intravenously and the serum was collected at 30 min (n = 6 mice per group). Data were means and error bars represented SEM. **c**, Left, OCR measurement. OCR was measured following the treatment with Oxamate (10 mM, 6 hr), GSK2837808A (20 μM, 6 hr) or Gefitinib (100 nM, 24 hr) using Seahorse XF96 analyzer. Right, OCR change fold. The OCR values were normalized by the control group without treatment (CON). The assays were performed in biological four replicates, and error bars represented SD. **d**, Immunohistochemistry analysis of representative tumor tissues from NSCLC PDX models with *FGFR* gene alteration or wildtype RTK. Shown are representative fields from one section per tumor tissue (n = 3 independent tumor tissues from each PDX model). Scale bar, 20 μm. **e**, Dependency score for metabolic

genes in cell lines with (Amp) or without (WT) *FGFR* amplification. Data were extracted from public dataset Project Achilles. $^{*}p < 0.05$ was considered to be statistically significant. **f**, Left: tumor growth curve of NCI-H1581 xenograft model. Mice bearing NCI-H1581 tumors were treated with Oxamate (750 mg/kg) and AZD4547 (2.5 mg/kg) daily for 21 days (n = 5). Growth curve was plotted by measuring the tumor volume three times per week. Right: Body weight change of NCI-H1581 and SNU16 xenograft models. Data were means and error bars represented SEM. **g**, Body weight of LU6429 PDX model. Oxamate (750 mg/kg), Metformin (250 mg/kg), AZD4547 (10 mg/kg) or indicated combinations were given daily (n = 6 except for Oxamate-treated group n = 7). Data were means and error bars represented SEM. **h**, Tumor growth curve, grouped scatter plot of individual mice relative tumor volume on Day 24 and body weight change of LU0743 PDX model. Mice were treated with Oxamate (750 mg/kg), AZD4547 (5 mg/kg) or indicated combination daily for 32 days (n = 6). Growth curve was plotted by measuring the tumor volume three times per week. Data were means and error bars represented SEM. **i**, Diagram depicting the metabolic reprogramming upon FGFR activation. **j-k**, Tumor

growth curve, grouped scatter plot of individual mice relative tumor volume at the endpoint, and body weight change of A431 xenograft and three PDX models. Mice bearing indicated tumors were treated with Oxamate (750 mg/kg) or NCT503 (40 mg/kg) daily for indicated days (n = 10 for A431, n = 5 for LU2071, n = 6 for LU-01-0393 and LU-01-0416). Data were means and error bars represented SEM. For all bar graphs, $^{***}p < 0.001$, $^{**}p < 0.01$, $^{*}p < 0.05$, n.s. ≥ 0.05 for Student's pairwise t test. Source data are provided as a Source Data file.

Supplementary Figure 4

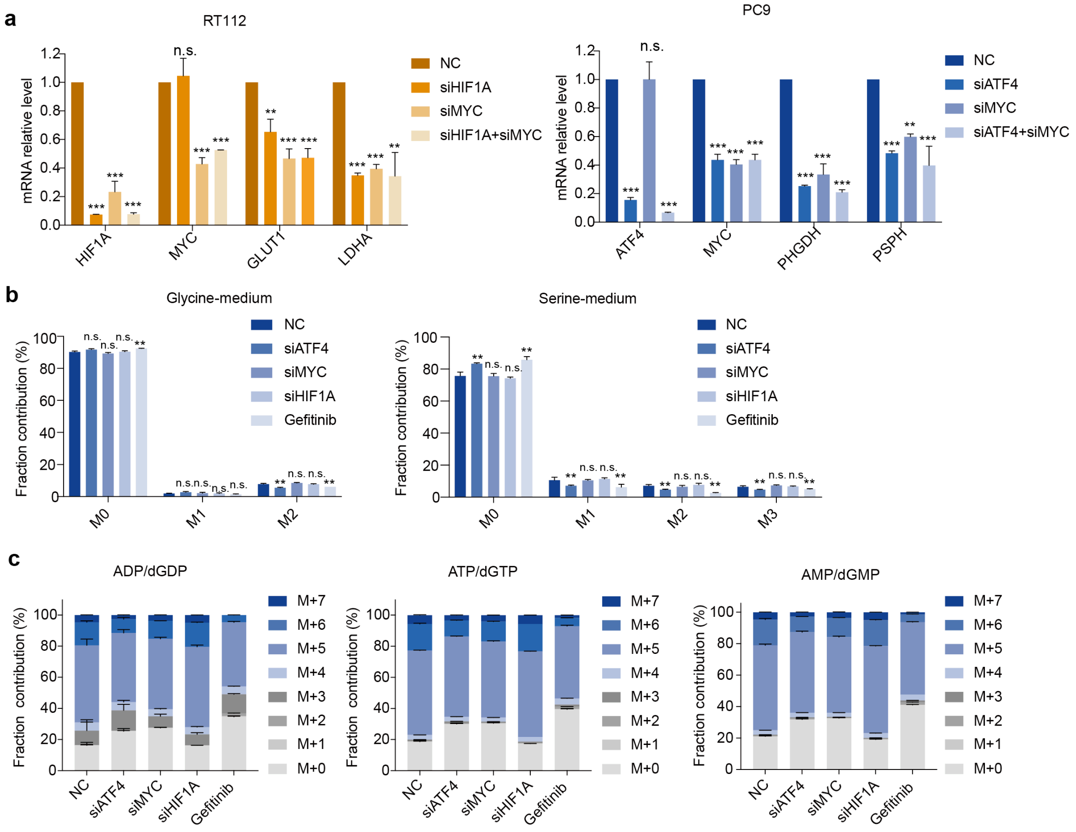

Supplementary Figure 4

a, RT-qPCR transcript analysis. RT112 and PC9 cells were transfected with indicated siRNA for 48 hr and mRNA level of indicated genes was measured by RT-qPCR. Data were means of triplicate wells; error bars represented SD. **b**, Analysis of glucose-derived serine and glycine in the medium of PC9 cells. Cells were transfected with indicated siRNA for 48 hr followed by 24 hr-culture in the presence of [U-$^{13}C_6$]-glucose. Serine and glycine were measured by GC/MS. Gefitinib treatment (100 nM, 24 hr) was used as a positive control. **c**, Analysis of glucose-derived purine nucleosides in PC9 cells. Cells were treated as in **b** and purine nucleoside was measured by QTOF-MS. Data in **b-c** were means of triplicates; error bars represented SEM. For all bar graphs, $^{***}p < 0.001$, $^{**}p < 0.01$, $^{*}p < 0.05$, n.s. ≥ 0.05 for Student's pairwise t test. Source data are provided as a Source Data file.
