# Supplementary material for: Identification of metabolic vulnerabilities of receptor tyrosine kinases-driven cancer
Source: Nat Commun. 2019 Jun 20;10:2701. doi: 10.1038/s41467-019-10427-2 (PMC6586626; doi:10.1038/s41467-019-10427-2)
Supplement: Supplementary file 3 — Reporting Summary [file 41467_2019_10427_MOESM3_ESM.pdf]

nature research

Corresponding author(s): Meiyu Geng, Shu-Hai Lin, Min Huang

Last updated by author(s): Apr 11, 2019

Reporting Summary

Nature Research wishes to improve the reproducibility of the work that we publish. This form provides structure for consistency and transparency in reporting. For further information on Nature Research policies, see Authors & Referees and the Editorial Policy Checklist.

Statistics

For all statistical analyses, confirm that the following items are present in the figure legend, table legend, main text, or Methods section.

n/a	Confirmed	
☐	☒	The exact sample size (*n*) for each experimental group/condition, given as a discrete number and unit of measurement
☐	☒	A statement on whether measurements were taken from distinct samples or whether the same sample was measured repeatedly
☐	☒	The statistical test(s) used AND whether they are one- or two-sided<br>*Only common tests should be described solely by name; describe more complex techniques in the Methods section.*
☒	☐	A description of all covariates tested
☒	☐	A description of any assumptions or corrections, such as tests of normality and adjustment for multiple comparisons
☐	☒	A full description of the statistical parameters including central tendency (e.g. means) or other basic estimates (e.g. regression coefficient) AND variation (e.g. standard deviation) or associated estimates of uncertainty (e.g. confidence intervals)
☐	☒	For null hypothesis testing, the test statistic (e.g. *F*, *t*, *r*) with confidence intervals, effect sizes, degrees of freedom and *P* value noted<br>*Give P values as exact values whenever suitable.*
☒	☐	For Bayesian analysis, information on the choice of priors and Markov chain Monte Carlo settings
☒	☐	For hierarchical and complex designs, identification of the appropriate level for tests and full reporting of outcomes
☒	☐	Estimates of effect sizes (e.g. Cohen's *d*, Pearson's *r*), indicating how they were calculated

Our web collection on statistics for biologists contains articles on many of the points above.

Software and code

Policy information about availability of computer code

Data collection	1) For lung adenocarcinoma analysis, LUAD dataset was extracted from TCGA database and used for further analysis.<br>2) To identify key TFs regulating metabolic DEGs, 1118148 TF-targets interaction data points for 2160 TFs were collected from public databases including Cistrom, mSigDB, OregAnno, CellNet and UCSC.<br>3) To reveal the cell growth dependency on specific metabolic genes, Project Achilles, a CRISPR/Cas9 screening-based dataset, was used for further analysis.
Data analysis	1) For the analysis of TCGA patients, gene functions were enriched by R package ClusterProfiler using annotation of GO-Biological Process. Gene amplifications were annotated by the cBioPortal public dataset through GISTIC algorithm.<br>2) The transcription factors (TFs) were enriched using Fisher's exact test and p-values were adjusted using Benjamini-Hochberg method. Finally, all TFs with adjusted p < 0.0005 were selected to be key TFs regulating metabolic DEGs. All these procedures were implemented in R 3.4.2.<br>3) The algorithm used to analyze the CRISPR data is CERES.

For manuscripts utilizing custom algorithms or software that are central to the research but not yet described in published literature, software must be made available to editors/reviewers. We strongly encourage code deposition in a community repository (e.g. GitHub). See the Nature Research guidelines for submitting code & software for further information.

Data

Policy information about availability of data

All manuscripts must include a data availability statement. This statement should provide the following information, where applicable:

- Accession codes, unique identifiers, or web links for publicly available datasets
- A list of figures that have associated raw data
- A description of any restrictions on data availability

All the data supporting the findings of this study are available within this paper and its supplementary information.

Field-specific reporting

Please select the one below that is the best fit for your research. If you are not sure, read the appropriate sections before making your selection.

☒ Life sciences ☐ Behavioural & social sciences ☐ Ecological, evolutionary & environmental sciences

For a reference copy of the document with all sections, see nature.com/documents/nr-reporting-summary-flat.pdf

Life sciences study design

All studies must disclose on these points even when the disclosure is negative.

Sample size	Sample-size calculations were not required for experiments of this study. Experiments were independently repeated or carried out in biological replicates. The exact sample size (n) for each experimental group/condition was provided, and mean/the standard error were calculated in the manuscript.
Data exclusions	No data were excluded from the analysis.
Replication	All attempts at replication were successful. Experiments were carried out in biological replicates as indicated in the manuscript.
Randomization	In this paper, the mice bearing tumors (including PDX models and xenograft models) were randomized into groups and treated with vehicle or indicated drugs.
Blinding	Blinding was not used in this study.

Reporting for specific materials, systems and methods

We require information from authors about some types of materials, experimental systems and methods used in many studies. Here, indicate whether each material, system or method listed is relevant to your study. If you are not sure if a list item applies to your research, read the appropriate section before selecting a response.

Materials & experimental systems

n/a	Involved in the study	
☐	☒	Antibodies
☒	☐	Eukaryotic cell lines
☒	☐	Palaeontology
☐	☒	Animals and other organisms
☒	☐	Human research participants
☒	☐	Clinical data

Methods

n/a	Involved in the study	
☒	☐	ChIP-seq
☒	☐	Flow cytometry
☒	☐	MRI-based neuroimaging

Antibodies

Antibodies used	The following antibodies were used: FGFR1 (9740S), p-EGFR Tyr1068 (3777S), EGFR (4267S), p-MET Tyr1003 (3135S), MET (8198S), p-RET Tyr905 (3221S), RET (3223S), p-AKT Ser473 (4060L), AKT (4691S), p-ERK1/2 Thr202/Tyr204 (4370L), ERK1/2 (4695S), p-STAT3 Tyr705 (9145S) and STAT3 (9139S) from Cell Signaling; p-FGFR1 Tyr653/654 (06-1433) from Millipore; PHGDH (14719-1-AP), PSPH (14513-1-AP) and GAPDH (60004-1) from Proteintech; β-actin (P30002) from Abmart.
Validation	Documents certify that the products have met the quality control standards defined by the manufactures.

Animals and other organisms

Policy information about studies involving animals; ARRIVE guidelines recommended for reporting animal research

Laboratory animals	Species: SCID mice, NU/NU nude mice; Sex, Female; Age, 4-6 weeks
Wild animals	This study did not involve wild animals.
Field-collected samples	This study did not involve field-collected samples.
Ethics oversight	The experiments of A431, SNU16 and NCI-H1581 xenograft models were approved and performed according to the Institute Animal Care and Use Committee (IACUC) at Shanghai Institute of Materia Medica. The experiments of PC9 xenograft, LU-01-0251, LU-01-0393 and LU-01-0416 PDX models were approved and performed according to the IACUC at WuXi AppTec. The experiments of LU2071, LU0743 and LU6429 PDX models were approved and performed according to the IACUC at

CrownBio. During all the studies, the care and use of animals were conducted in accordance with the regulations of the Association for Assessment and Accreditation of Laboratory Animal Care (AAALAC).

Note that full information on the approval of the study protocol must also be provided in the manuscript.
