# Supplementary material for: Identification of metabolic vulnerabilities of receptor tyrosine kinases-driven cancer
Source: Nat Commun. 2019 Jun 20;10:2701. doi: 10.1038/s41467-019-10427-2 (PMC6586626; doi:10.1038/s41467-019-10427-2)
Supplement: Supplementary file 4 — Description of Additional Supplementary Files [file 41467_2019_10427_MOESM4_ESM.doc]

**Title: Supplementary Dataset 1.
Description:** List of growth inhibition rate of a panel of lung cancer cell lines to metabolism-targeted inhibitors. Cell growth inhibition rate was measured using CCK-8 assay after treatment with ETO (50 μM), CB839 (10 μM), Oxamate (30 mM), DCA (10 mM) or 2DG (2.5 mM) for 72 hr. The growth inhibition rate was obtained as normalized by the untreated group.

**Title: Supplementary Dataset 2.**
**Description:** List of the metabolites intensities in the metabolomics data of BAF3 and BAF3-RTK cells. Cells were subjected to GC/MS and UHPLC-QTOF-MS analysis.

**Title: Supplementary Dataset 3.**
**Description:** List of the enriched metabolite pathways obtained by the analysis of altered metabolites in BAF3-RTK cells (1.5-fold cutoff in relative to parental BAF3 cells; p < 0.01) using MetaboAnalyst 4.0.

**Title: Supplementary Dataset 4.
Description:** List of the fractional contribution of ^13^C-labeled metabolite isotopologues in BAF3 and BAF3-RTK cells. Cells were cultured in the presence of [U-^13^C_6_]-glucose (12 hr), [U-^13^C_5_]-glutamine (24 hr) or [U-^13^C_16_]-palmitate (24 hr) prior to mass spectrometry analysis.

**Title: Supplementary Dataset 5.**
**Description:** The information of all the cells lines used in the study including vendor, catalogue number, culture condition, histological type and identified genotype.

**Title: Supplementary Dataset 6.**
**Description:** List of enriched pathways identified using KEGG pathway enrichment analysis of differentially transcribed clusters in the heatmap of transcriptome profiling (Supplementary Fig. 1**p**) of BAF3 and BAF3-RTK cells.

**Title: Supplementary Dataset 7.
Description:** The information of all the PDXs used in the study including histological type, identified genotype and vendor.

**Title: Supplementary Dataset 8.
Description:** List of identified transcriptional factors (TFs) that orchestrate metabolic reprogramming using bioinformatics analysis. TFs were identified by establishing a network model describing the TF-target interactions according to the published TF databases (Cistrom, ORegAnno, mSigDB, CellNet and UCSC).

**Title: Supplementary Dataset 9.**
**Description:** List of metabolic genes expression change upon knockdown of transcriptional factors (TFs). Cells were transfected with siRNAs targeting representative TF identified in Supplementary Dataset **8** for 72 hr and metabolic gene expression alteration was measured by RT-qPCR. The expression level of indicated genes was normalized by that of the untreated group.
